# Performance and application of the total-body PET/CT scanner: a literature review

**DOI:** 10.1186/s13550-023-01059-1

**Published:** 2024-04-12

**Authors:** Yuanyuan Sun, Zhaoping Cheng, Jianfeng Qiu, Weizhao Lu

**Affiliations:** 1 Department of Radiology, Shandong First Medical University & Shandong Academy of Medical Sciences, Taian, 271016 China; 2 Department of PET-CT, The First Affiliated Hospital of Shandong First Medical University, Shandong Provincial Qianfoshan Hospital Affiliated to Shandong University, Jinan, 250014 China; 3 Department of Radiology, The Second Affiliated Hospital of Shandong First Medical University, No. 366 Taishan Street, Taian, 271000 China

**Keywords:** Total-body PET/CT, uEXPLORER, Performance evaluation, Clinical application, Deep learning

## Abstract

**Background:**

The total-body positron emission tomography/computed tomography (PET/CT) system, with a long axial field of view, represents the state-of-the-art PET imaging technique. Recently, the total-body PET/CT system has been commercially available. The total-body PET/CT system enables high-resolution whole-body imaging, even under extreme conditions such as ultra-low dose, extremely fast imaging speed, delayed imaging more than 10 h after tracer injection, and total-body dynamic scan. The total-body PET/CT system provides a real-time picture of the tracers of all organs across the body, which not only helps to explain normal human physiological process, but also facilitates the comprehensive assessment of systemic diseases. In addition, the total-body PET/CT system may play critical roles in other medical fields, including cancer imaging, drug development and immunology.

**Main body:**

Therefore, it is of significance to summarize the existing studies of the total-body PET/CT systems and point out its future direction. This review collected research literatures from the PubMed database since the advent of commercially available total-body PET/CT systems to the present, and was divided into the following sections: Firstly, a brief introduction to the total-body PET/CT system was presented, followed by a summary of the literature on the performance evaluation of the total-body PET/CT. Then, the research and clinical applications of the total-body PET/CT were discussed. Fourthly, deep learning studies based on total-body PET imaging was reviewed. At last, the shortcomings of existing research and future directions for the total-body PET/CT were discussed.

**Conclusion:**

Due to its technical advantages, the total-body PET/CT system is bound to play a greater role in clinical practice in the future.

## Background

Positron emission tomography (PET) is a molecular imaging technique, and an important diagnostic imaging approach in nuclear medicine [1–4]. PET can detect changes in the physiological activity of lesions before morphological alterations occur using different tracers [3]. Combined with structural imaging modalities such as magnetic resonance imaging (MRI) or computed tomography (CT), PET has played critical roles in the assessment of tumors, neurological disorders and cardiovascular diseases [5–7].

Since the introduction of the first commercial PET in 1976, PET systems have developed rapidly [1]. The core of the PET development is essentially the development of detectors [8]. A dense and fast-decaying scintillator, a highly sensitive photo-sensor, as well as precise custom-designed readout electronics are the three key components of the detector blocks [8–14]. The developments of these technologies have transformed PET from the clinical need to capture a single organ to the need to link multiple organs in vivo [1]. However, limited by the axial field of view (aFOV) of traditional PET scanners, multi-bed positions are necessary to cover the patient’s whole torso (imaging “from eyes to thighs”) during image acquisition [8], which may result in quantification inaccuracies due to differences in the acquisition time and noise levels [15]. Henceforth, reducing the number of imaging bed positions and even achieving whole-body imaging has been the trend of PET development [1]. More importantly, the concept of whole-body imaging has large potential with regard to low-dose imaging, faster scanning, whole-body dynamic imaging and follow-up of tracers over longer periods, which may change the current clinical routine and expand the number of clinical applications of molecular imaging [7]. The pursuit of total-body imaging advances the aFOV of the detector. PET system reached aFOVs of more than 20 cm in the second half of the 2000s [16, 17]. In the late 2010s, the aFOV of the new generation PET scanner has developed to above 1 m [16, 17]. These scanners allow total-body acquisition within less than 60 s and thereby will give a real-time insight into (patho-)physiological processes [8].

Currently, long aFOV (> 50 cm) PET scanners are commercially available worldwide [18], and three human total-body PET/CT systems have been developed: the PennPET Explorer, the uEXPLORER, and the Biograph Vision Quadra [8]. These three PET systems all realize aFOVs above 1 m and show a tremendous increase in system sensitivity due to the longer gantries [8]. The PennPET Explorer, with recently-extended aFOV of 1.12 m and a time-of-flight (TOF) resolution of 250 ps, enables imaging the entire human body with a limited number of bed positions (2–3 bed positions) [18]. The Biograph Vision Quadra has an aFOV of 106 cm, offering anatomical coverage roughly from head to thighs [19]. Among the three total-body PET systems, the uEXPLORER is the first real-sense total-body PET/CT with a total aFOV of 194.0 cm and offers uniform sensitivity throughout its one-meter length center [20].

Compared with traditional PET systems, the long aFOV makes the total-body PET/CT systems possible to simultaneously image most organs of interest in adults with a limited number of bed positions and uniform image quality [18–22]. The total-body PET/CT system enables superb spatial resolution, increased signal-to-noise ratio (SNR), and lesion detection capability, as well as low-dose and sub-second imaging [22–27]. As the total-body PET/CT has been increasingly used in research and clinical investigations, it is necessary to summarize its performance and clinical applications, with the aim to provide a reference for researchers and to point out its future directions. This review collects studies on total-body PET/CT from the PubMed database over the last 5 years using keywords including total-body PET, whole-body PET, PennPET Explorer, uEXPLORER, or Biograph Vision Quadra and collected 86 publications. The review is divided into the following sections: In “Main text” section, we gave a brief introduction of the total-body PET/CT system, reviewed the studies on performance evaluation of the total-body PET/CT, summarized the research and clinical applications of the total-body PET/CT, and summed up deep learning-based studies using the total-body PET/CT. In “Discussions and conclusions” section, we presented an outlook on the clinical applications of the total-body PET system based on the summary of the existing literatures.

## Main text

### A brief introduction to the total-body PET/CT system

**Fig. 1:**
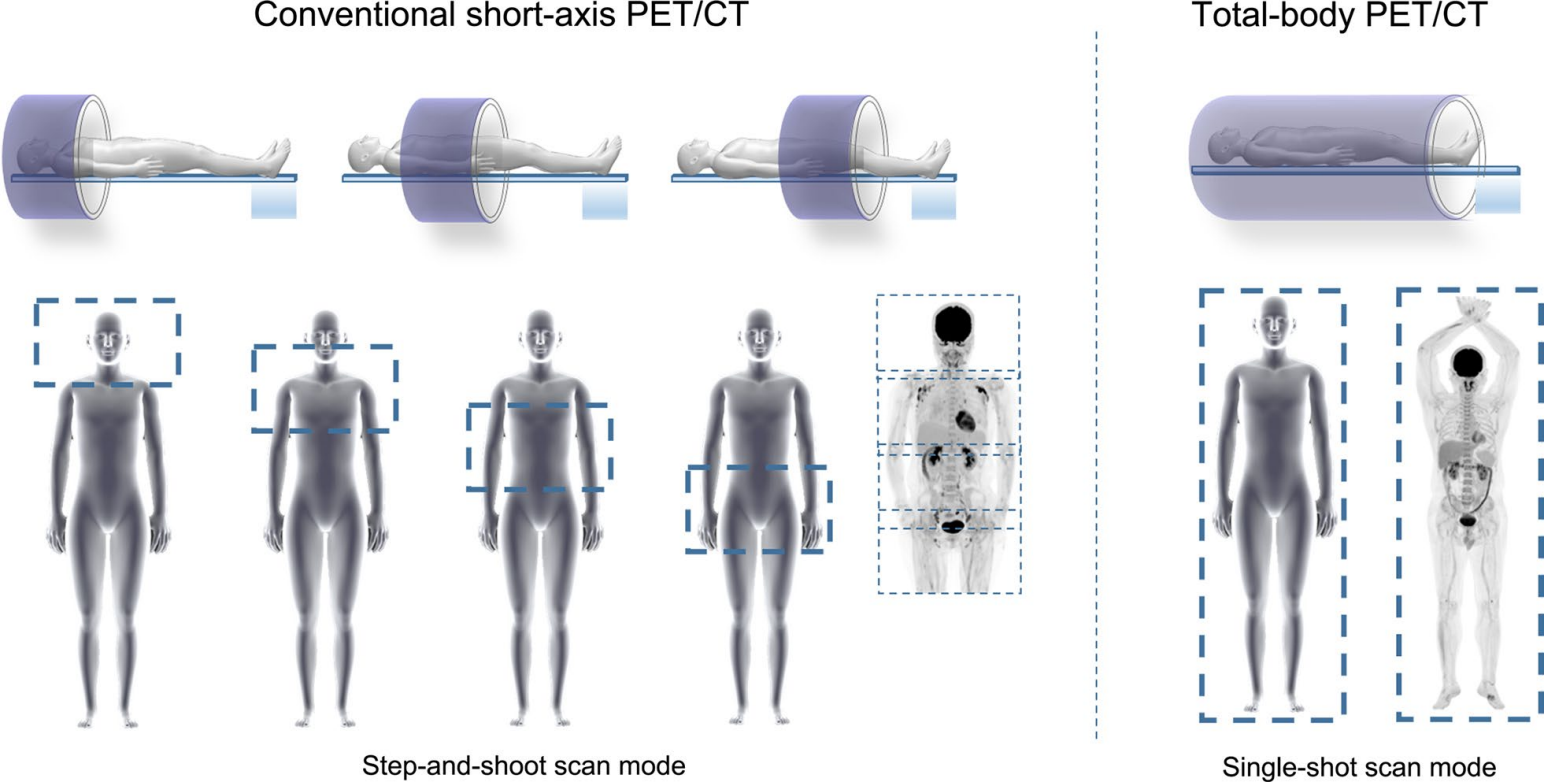
Total-body PET/CT versus conventional PET/CT

Compared to conventional PET, the total-body PET/CT system brings the state-of-the-art hardware enhancement, transforming the traditional 20–25 min step-and-shoot scan mode to single-shot whole-body scan (Fig. 1). The PennPET Explorer is composed of 6 ring segments, each ring covers a 22.9 cm-long aFOV with 16.4 cm of active detectors. The PennPET Explorer utilizes a detector tile of 64 lutetium-yttrium oxyorthosilicate (LYSO) scintillation crystals [18]. For the Biograph Vision Quadra, the total axial span of 320 LYSO crystals is directly coupled to a silicon photomultiplier array with 16 output channels. Eight mini blocks form a detector block, with 2 adjacent detector blocks sharing a common electronic unit. This arrangement enables the Biograph Vision Quadra an aFOV of 106 cm [28]. The uEXPLORER is composed of 8 PET units along the axial direction; each unit is 24.02 cm in axial length, 78.6 cm in diameter, with a 0.26 cm gap between units, making up the system with axial length of 194 cm. There are 24 detector modules in each PET unit, and each module contains 70 block detectors arranged in a 5 × 14 matrix [22].

### Performance evaluation of the total-body system

Theoretically, sensitivity gain provided by the long aFOV PET scanner will lead to significant improvements in system performance, which will benefit to low-dose imaging, delayed imaging, ultrafast imaging, and total-body dynamic imaging [29, 30]. This section gives a detailed overview of the basic performance evaluation and performance under various extreme conditions for different total-body PET/CT systems.

#### Basic performance of the total-body system

Studies have evaluated comprehensive performances of the three total-body PET/CT systems using metrics including sensitivity, count-rate performance, time-of-flight resolution, spatial resolution, and image quality based on the NEMA NU-2-2018 protocol [22, 28, 31], and the results are presented in Table 1. In summary, the three total-body PET/CT systems have good performances in terms of spatial resolution, time resolution and sensitivity. The uEXPLORER scanner provides a very high sensitivity, and the longest aFOV among the three systems [32]. However, the PennPET Explorer and Quadra have higher time resolution and peak noise equivalent count-rate (P-NECR) [25, 28].

**Table 1:** Major specifications and performance metrics of the three total-body PET/CT systems

Metrics	PennPET explorer	Quadra	uEXPLORER
*aFOV*	64 cm (prototype) 112 cm (upgraded)	106 cm	194 cm
Sensitivity (3D)	54 kcps/MBq	83.4 kcps/MBq (MRD 85) 176.0 kcps/MBq (MRD 322)	171 kcps/MBq
Space resolution@1 cm*	4.0 mm	3.8 mm	2.9 mm
Space resolution@10 cm*	–	–	3.2 mm
scatter fraction	32%	36% (MRD 85) 37% (MRD 322)	38%
P-NECR	1550 kcps@25 kBq/cc	1613 kcps@27.49 kBq/ml (MRD 85) 2956 kcps@27.49 kBq/ml (MRD 322)	1549 kcps@16.8 kBq/cc
Time resolution	256 ps	228 ps (MRD 85) 230 ps (MRD 322)	430 ps
Energy window range (3D)	450–630 keV	435–585 keV	430–650 keV
Data collection method	3D, List-mode, Static, Dynamic	3D, List-mode, Static, Dynamic	3D, List-mode, Static, Dynamic, Gating

*@1 cm means at the site 1-cm away from the imaging center, @10 cm means at the site 10-cm away from the imaging center

*MRD 85* maximum ring distance of only 85 crystals, *MRD 322* maximum ring distance of 322 crystals

#### Ultrafast imaging

**Fig. 2:**
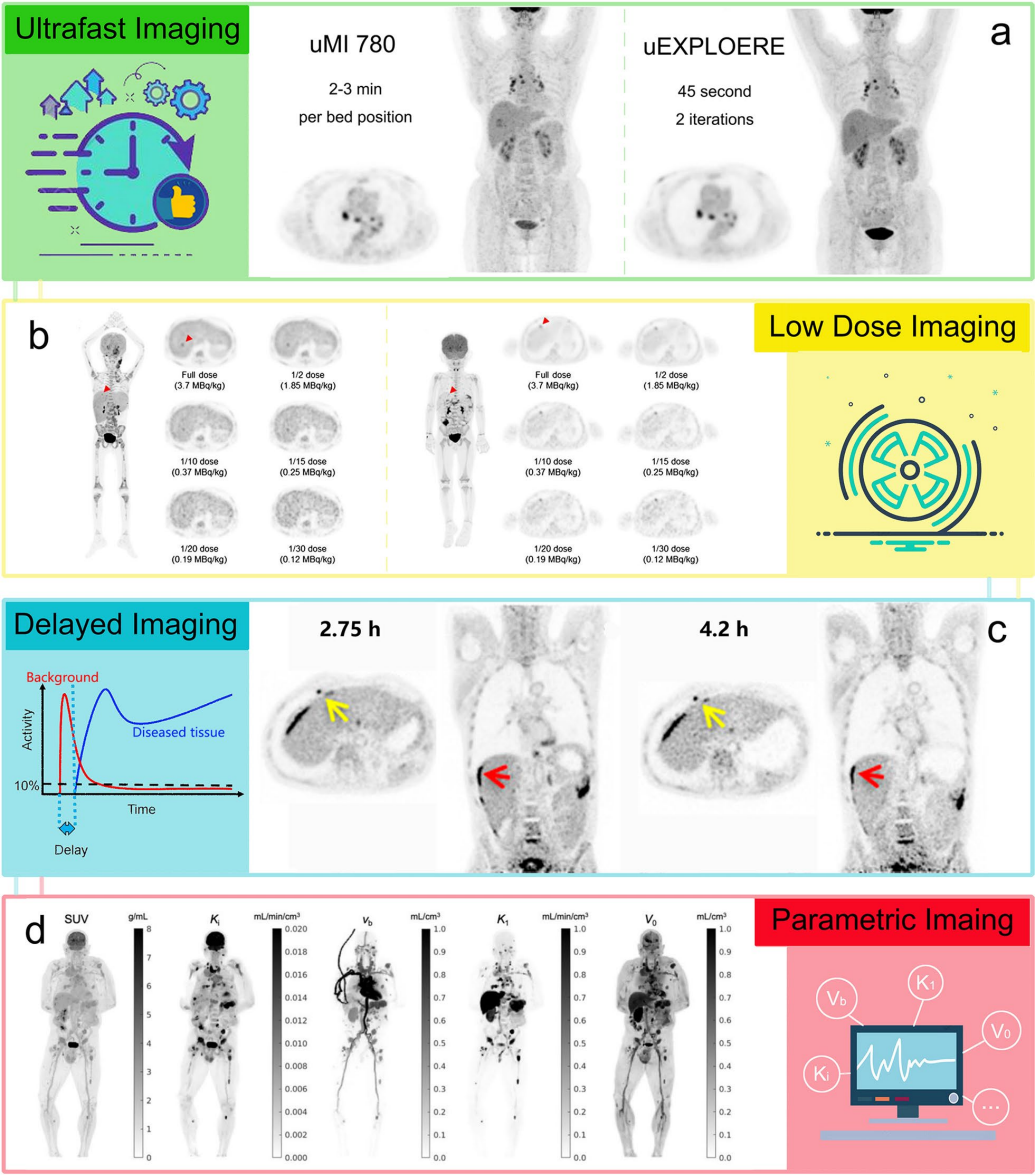
Performance evaluation of the total-body PET systems. **a** PET transverse and maximum intensity projection (MIP) images of a 64-year-old woman diagnosed with hepatocellular carcinoma. Left panels are reconstructed images with the clinical protocol via a conventional PET/CT scanner (uMI 780), and right panel is reconstructed images with 2 iterations and a 45-s acquisition via the uEXPLORER. The protocol with a 45-s scanning duration and 2 iterations using the uEXPLORER can provide equivalent image quality as the uMI 780 [35]. **b** MIP of the full-dose image and axial view of the serial dose reduction images generated by reduced count. Left: An FDG-avid micro-lesion in the liver of a 7-year-old patient with neuroblastoma, with an SUV_max_ of 4.35 on the full-dose image. The lesion is identifiable as reduced down to 1/20-dose and is un-diagnosable at 1/30-dose. Right: A micro-lesion in the subcapsular region of the liver in a 3-year-old patient with Burkitt Lymphoma is diagnosable in all dose reduction images [41]. **c** Dynamic scan is acquired after the injection of 496 MBq of ^18^F-FDG on a 60-year-old patient with metastatic colon cancer. Panels are 10-min reconstructions at 2.75- and 4.2-h post-injection using the PennPET scanner, respectively [33]. **d** Comparison of standard SUV image with parametric images of FDG influx rate *K*_*i*_, fractional blood volume *V*_b_, FDG delivery rate *K*_1_ and volume of distribution *V*_0_ images of a cancer patient. The images are shown as MIP maps [54]. It is worth mentioning that due to the different window widths, tumors may be more apparent in the parametric map than in the SUV map. In fact, there is no difference in tumor numbers between the two set of images

Owing to the increased sensitivity of long aFOV, the total-body PET scanners can generate diagnostic level images with reduced scan time. Researchers simulated the effects of decreased scan time using list-mode data and the results demonstrated that the PennPET Explorer and Quadra can generate images of satisfactory quality in 2 min with comparable SNR and lesion detectability to routine clinical images [18, 19, 26, 33], while the uEXPLORER can generate images with acceptable image quality in 30 s with SNR and lesion detectability similar to routine clinical images [34]. In addition, the Quadra and PennPET Explorer PET/CT systems could deliver images of comparable quality and lesion quantification in under 2 min, compared to routine clinical scan of 16 min from short aFOV PET/CT scanner [26]. Previous studies demonstrated that a protocol with 30–45-s scanning duration and 2 or 3 iterations for the uEXPLORER was found to provide an equivalent image quality as the traditional imaging protocol for the uMI 780 (a clinical 3.0 T MRI scanner from the United Imaging), while the former protocol showed higher SNR for the lesions (Fig. 2a) [35], and 60-s images by the uEXPLORER were still feasible in oncological applications, as all the lesions could be identified in 60-s images [36].

#### Low-dose imaging

The increased sensitivity of the total-body PET allows reduced dose of the injected radiopharmaceuticals while maintaining acceptable image quality. For Quadra PET/CT system, low-dose (2.0 MBq/kg) ^18^F-FDG with reduced scan time up to 5 min provided comparable data to standard acquisition in melanoma diagnose [37]. A phantom and lesion embedding study with ^18^F-fluorothymidine on the PennPET Explorer has shown the ability to accurately estimate kinetic parameters, with doses as low as 0.5–2 mCi [38].

PET images from the uEXPLORER with 1/20 of the standard injection dose (18.5 MBq/0.5 mCi) are still considered to be of high quality for clinical use [34]. According to the study by Tan et al., image quality scores, liver SNR, and lesion detectability of PET images with 2-min acquisition time and half-dose (1.85 MBq/kg) by the uEXPLORER were significantly higher than those with full dose by conventional PET scanner [39]. Later, Tan et al. also investigated lesion detectability in colorectal cancer patients at ultra-low dose (0.37 MBq/kg ^18^F-FDG). The study proved that PET images with ultra-low dose and the acquisition time of 1-min was sufficient for clinical diagnosis and lesion detectability, although the image quality was degraded [40]. Zhao et al. simulated low-dose (1/30–1/2 dose, 0.12–1.85 MBq/kg) images by truncating the list-mode PET data to reducing count density and compared them with full-dose images (3.70 MBq/kg). Results showed that sufficient subjective image quality and lesion conspicuity could be maintained down to 1/30-dose (0.12 MBq/kg) of the administered dose of ^18^F-FDG, where good image quality scores were given between 1/2- and 1/10-dose (0.375–1.85 MBq/kg) groups (Fig. 2b) [41].

#### Delayed imaging

In addition to imaging with less injected dose, the high sensitivity of the total-body PET/CT system enables imaging at delayed time points [34]. Delayed imaging 24 h after injection of ^18^F-FDG (half-life 110 min) in humans has been proved to be feasible on the PennPET Explorer [33], and even more delayed imaging can be obtained for longer-lived radiotracers, such as ^89^Zr (half-life 3.3 days), to study slower biologic processes [18]. As for the uEXPLORER, depending on radiotracer kinetics, delayed imaging may improve contrast in diseased tissues such as atherosclerosis and cancers (Fig. 2c) [42, 43]. Hu et al. tested delayed imaging for different organs in 10 healthy volunteers with ^18^F-FDG. In vivo time-activity curves (TACs) of all investigated organs acquired using standard imaging method (up to 75-min post-injection) and delayed imaging method (up to 8-h post-injection) were compared. It was found that the average residence time differences of the brain, heart, kidney, liver, and lungs were 8.38%, 15.13%, 25.02%, 23.94%, and 16.50% between standard imaging delayed imaging methods. In addition, the bladder revealed the largest difference (21.18%) in the effective dose between the two imaging methods among all the investigated organs [44].

#### Total-body dynamic imaging and parametric imaging

The ultra-high time resolution and total-body coverage of the total-body PET/CT systems pave the way for total-body dynamic imaging, and enhance the image quality of parametric imaging [34, 45]. Rausch et al. [19] demonstrated the feasibility of dynamic imaging for Biograph Vision Quadra PET/CT system with 10-min dynamic imaging of phantoms. Later, van Sluis et al. [46] generated ^18^F-FDG net influx rate (Ki) images of 12 patients with suspected lung malignancy from a 65-min dynamic PET acquisition using the Quadra PET/CT system and 2 reconstruction settings and demonstrated good agreements between the two reconstruction approaches. Larsson et al. [47] estimated cerebral blood flow of 25 patients from a 60-min dynamic PET imaging using the Quadra PET/CT system and model‑free deconvolution approach. Li et al. [48] developed a deep learning approach to generate parametric images directly from the sinograms using Quadra PET/CT system, and the results demonstrated that high-quality Patlak Ki images could be generated based on the deep learning approach with high structural similarity and increased peak SNR. As for the PennPET Explorer, ^18^F-fluortriopride and 30-min dynamic scan was performed for one subject, with images centered over the gallbladder. Representative images (1-min scans) demonstrated mild gallbladder emptying over time, underscoring the potential of the PennPET Explorer for dynamic imaging of multiple organs [18].

In terms of the uEXPLORER, Zhang et al. [49] developed a method to perform ultra-high temporal resolution dynamic PET imaging and visualized radiotracer transport across the entire body on timescales of 100-ms and obtained motion-frozen images with higher image quality compared to traditional methods. Sun et al. [50] demonstrated a frame-by-frame correction framework that can effectively reduce the effect of random body movements on dynamic images and the associated quantification process with overall quality improvement in quantitative Ki images. Wang et al. [51] developed a dual-time-window protocol based on the uEXPLORER, and reduced dynamic scanning time from 60 min to less than 20 min without affecting the diagnostic efficacy. The uEXPLORER also allows high-quality parametric imaging across the entire body, which can better characterize heterogeneous regional tracer kinetics than ROI-based analysis [52]. Zhang et al. developed quantitative parametric image reconstruction methods for kinetic analysis via uEXPLORER and demonstrated good image quality of dynamic images with low noise, even for the 1-s frames. In addition, there were excellent delineations of the smaller structures in cerebral, cardiac and vertebral regions [53]. Wang et al. performed 60-min total-body dynamic PET imaging for cancer patients and used voxel-wise compartmental model for parametric image reconstruction. Results showed improved lesion TAC fitting, physiologically more consistent vasculature in the fractional blood volume (*v*_b_) image, and improved tumor contrast (Fig. 2d) [54]. In addition, parametric images can be potentially more useful than standardized uptake value (SUV) for liver tumor imaging and brain tumor imaging, as lesions are more visible with higher contrast on the Ki image than the SUV image [54].

In summary, despite the advantages of total-body PET systems in ultrafast, low-dose, and delayed imaging, combining these methods simultaneously may result in a decrease in image quality. Appropriate methods should be selected based on clinical needs, such as fast imaging for breath-holding lung imaging, low-dose imaging for children, and healthy individuals. While delayed imaging may require a higher radiation injection dose, which deserves careful consideration.

### Research application of the total-body PET/CT systems

The advantages of the total-body PET/CT systems promise the applications [55]. Currently, research applications of the total-body PET/CT systems mainly involve tumor, nervous system, cardiovascular disease, and systemic immune diseases.

#### Application on the healthy participants

Due to radiation exposure, traditional PET/CT examination is generally used in the detection of tumors, and is not recommended for healthy individuals. However, the low-dose and ultra-low-dose imaging enabled by the total-body PET/CT systems have advanced the exploration of PET imaging in healthy individuals [56].

**Fig. 3:**
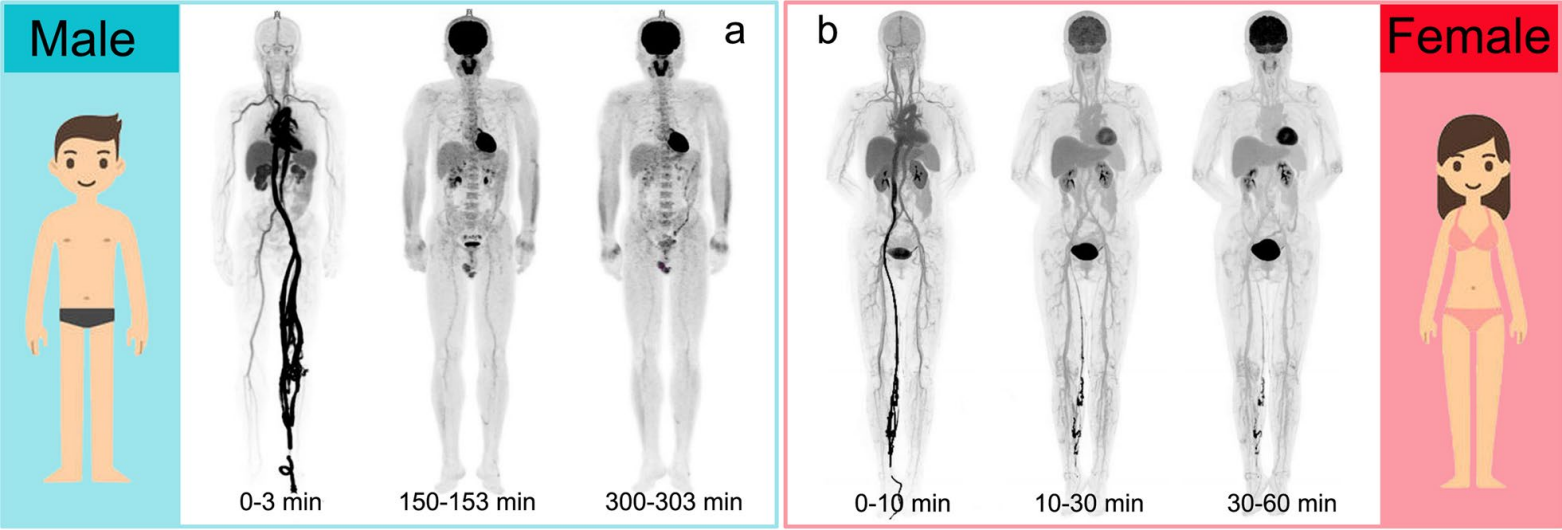
Dynamic PET/CT images of healthy volunteers. **a** Reconstructed dynamic PET images of early 3 min, 150–153 min, 300–303 min from a healthy male subject during and following an intravenous injection of ^18^F-FDG using a dosing regimen of 1.85 MBq/kg [44]. **b** Reconstructed dynamic composite SUV images of early 10-min, mid 20-min, late 30-min scans of a healthy female subject during and following an intravenous injection of 4.57 MBq/kg of ^18^F-FDG [53]. Dynamic PET/CT images from different sexes are for display purpose only

In 2020, Pantel et al. [18] demonstrated the ability to scan for a shorter duration using the PennPET Explorer scanner on healthy participants. Later, Zhang et al. [53] demonstrated the first total-body parametric imaging on a healthy volunteer via the uEXPLORER and demonstrated the spatiotemporal distribution of radiotracers in vivo in major organs/tissues (left ventricle, aorta, carotid artery, brachial artery, and femoral artery, myocardium, liver, gray matter, and white matter) (Fig. 3). Wang et al. [54] performed a 1-h dynamic scan after the injection of 370 MBq of ^18^F-FDG on 5 healthy volunteers via the uEXPLORER and successfully conducted total-body PET multiparametric imaging and precise quantification of different organs using voxel-wise compartmental modeling strategies. Nardo et al. [42] performed dynamic scan after the injection of 370 MBq of ^18^F-FDG on a 51-year-old healthy volunteer. The results showed significant reduction in blood pool activity at 3-h post-injection.

With increasing experience from total-body PET studies, uptake in normal physiologic structures becomes more prominent. For example, via total-body ^18^F-FDG imaging, the adrenal glands, pituitary gland, and gray matter of the spinal cord are prominent [57]. Recently, Derlin et al. investigated vessel wall biology of healthy volunteers using the uEXPLORER with ultra-low-dose (0.185 MBq/kg) ^18^F-FDG. Cross talk between vessel wall and lymphoid organs was identified with superior accuracy in total-body PET images, which might stimulate future mechanistic study or be used as biomarkers for monitoring vessel wall pathologies [58]. Lu et al. [59] demonstrated the in vivo glucose uptake and distribution across the human skeleton based on ^18^F-FDG total-body PET scan using the uEXPLORER and further revealed that skeletal glucose uptake can be affected by age and dysregulated metabolism.

#### Application in oncology

The technical advantages of the total-body PET systems are beneficial to oncology studies. For example, with extended aFOV compared to short-axis PET scanner, the total-body PET system provides a platform to locate tumors across the entire body and evaluate tumor metastasis [60]. The high sensitivity offers better TACs of organs, providing a good tool to study tumor kinetics [49, 61].

##### Tumors

**Fig. 4:**
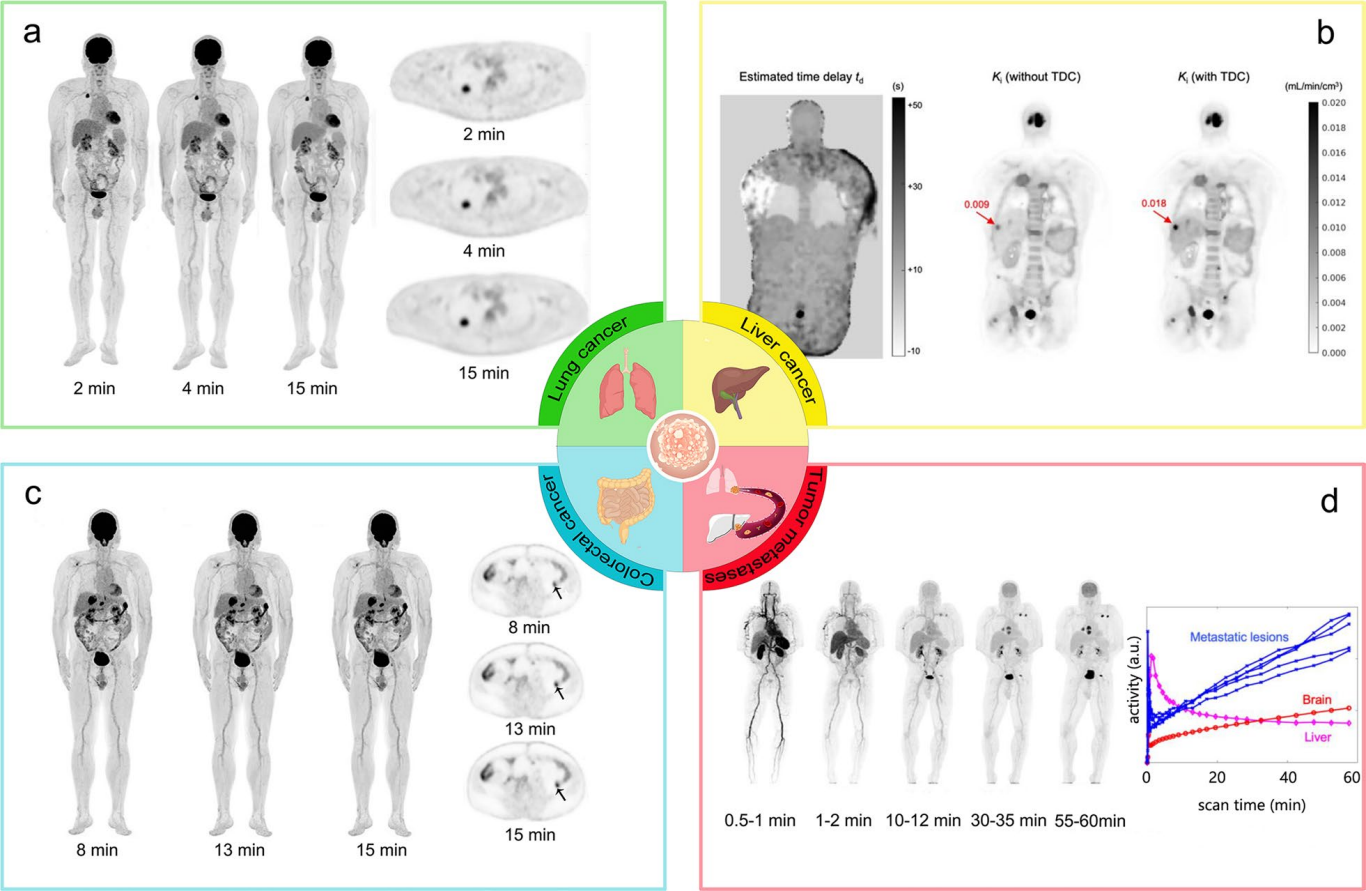
Total-body PET images of different types of cancers. **a** Total-body PET images of a 58-year-old man with infiltrating adenocarcinoma of the lung confirmed by surgery. The FDG-avid lesion in the upper lobe of the right lung is identified in MIP and axial images with different reconstruction times [39]. The times in the panel represent reconstruction duration. **b** Coronal view of the estimated time delay *t*_d_ map and *K*_*i*_ parametric images with and without time delay correction (TDC) for a patient with liver cancer. Arrows point to the liver cancer [54]. To better illustrate the lesions in the liver, the authors only demonstrate the upper part of the total-body PET images. **c** MIP maps with different reconstruction times of a 58-year-old male with adenocarcinoma of the splenic flexure of the colon diagnosed by pathological examination. The corresponding axial images show a low-grade intraepithelial neoplasia lesion in the descending colon [40]. The times in the panel represent reconstruction duration. **d** Total-body dynamic FDG PET images of a patient with metastatic renal cancer. The MIP images demonstrate higher renal FDG uptake in the late phase than that in the early phase [54]. The times in the panel represent scan time

At present, total-body PET/CT scanners have been applied in the research of cancers in different organs, including the lung, liver, gut, and urogenital system. Figure 4 summarizes the clinical application of total-body PET/CT systems in cancer research.

In terms of lung cancer, studies with both static and dynamic ^18^F-FDG PET acquisition of patients with lung cancer using the Quadra PET/CT demonstrated that images with reduced scan time did not exhibit substantial loss of accuracy and precision [46, 62]. Tan et al. [39] conducted half-dose (1.85 MBq/kg) and full-dose (3.70 MBq/kg) total-body PET scan of lung cancer patients, and demonstrated 100% lung cancer (primary and metastasis) detection rate via 2-min half-dose imaging (Fig. 4a). Sun et al. [63] conducted network analysis at the systemic and organ level between lung cancer patients and healthy controls, and showed network deviations of lung cancer patients from the reference network.

As for liver cancer, a standard ^18^F-FDG imaging protocol of 10 min was used via the Quadra scanner for oncological patients including recurrent hepatic cancer patient and scans were reconstructed at different time. The results showed improved lesion detection and classification while keeping the total scan time within 5 min [27]. Numerical simulations and preclinical scans both demonstrated clinically acceptable performance for detecting lesions in 1 min or less in the lung and liver via the prototype PennPET Explorer [33, 64]. A total-body ultrafast scan (30–45-s) of liver cancer patients showed that image quality and lesion tumor-to-background ratio of PET images from the uEXPLORER were not significantly different from that of uMI 780 via a standard clinical protocol (Fig. 4b) [35, 54].

For colorectal cancer, a qualitative comparison study showed ^18^F-FDG PennPET images to be of superior quality compared to traditional short-axis PET images with improvements in the delineation of sites of tumors for metastatic colon cancer when performed with similar scan durations [33]. An ultra-low-dose (0.37 MBq/kg) scan of colorectal cancer (CRC) patients via the uEXPLORER showed that the image quality of the 8-min group met the clinical diagnosis needs for CRC without affecting lesion detectability (Fig. 4c) [40].

In terms of genitourinary cancer, Quadra PET/CT scanner demonstrated improved image quality, lesion quantification, and SNR in prostate cancer scanning compared to short-axis PET scanner [26]. A 60-min total-body dynamic scan performed to genitourinary cancer patients following the injection of 370 MBq of ^18^F-FDG revealed all the lesions in SUV images. Improved TACs fitting, higher *K*_*i*_ in lesions were observed in multiparametric images [54].

In addition, total-body PET/CT systems have also been used in studies of lymphomas, melanomas and sarcomas. Both ultrafast scan and low-dose scan have been performed for patients with lymphomas and melanomas via the Quadra scanner, and results demonstrated improved image quality and lesion detectability due to the higher sensitivity of the long aFOV scanner [37, 62, 65, 66]. In the study by Chen et al., 100 pediatric oncological patients (including 9 infants) with lymphoma, rhabdomyosarcoma, and neuroblastoma underwent total-body PET/CT with half-dose (1.85 MBq/kg) ^18^F-FDG. The results illustrated sufficient image quality and lesion conspicuity of the low-dose imaging in the assessment of sarcomas and lymphomas [36]. The study by Tang et al. [67] applied the uEXPLORER PET/CT on 105 pediatric lymphoma patients, and the results showed excellent performances for the uEXPLORER in the diagnosis of nodal, splenic, bone marrow, and other extranodal lymphoma involvement.

##### Cancer of unknown primary

Cancer of unknown primary (CUP) is difficult to treat, because existing therapeutics are predominantly specific to the primary tumor [68, 69]. The total-body PET/CT scanner allows the assessment of multi-organ function (static PET imaging) and multi-organ interactions (dynamic PET imaging) across the entire body, which stands a chance to solve the issue of CUP [37, 70].

In a case report of a patient with lymph node metastasis of an unknown primary tumor, Lu et al. [60] used static total-body PET scan to localize possible locations with abnormal FDG uptake and used dynamic total-body PET scan to find the metabolic similarities between the metastatic tumor and possible primary tumor locations. In a subject with metastatic colon cancer, ^18^F-FDG PET imaging using the PennPET revealed an epiphrenic lymph node that was not obvious on clinical short-axis PET scan [33]. Wang et al. [54] found that in the same patient, multiple metastatic lesions showed similar TAC shapes and proved the value of dynamic imaging in the evaluation of metastatic tumors (Fig. 4d).

#### Other applications

The concept of tissue perfusion is linked with blood flow, oxygen delivery, and a combination of blood flow and nutrition supply. Monitoring of tissue perfusion is an essential step in the management of many diseases [71]. With the advent of total-body PET, quantitative measurement of perfusion across the entire body is possible. A previous study estimated brain perfusion with five tracers including ^15^O-H2O, ^11^C-PIB, ^18^F-FE-PE2I, ^18^F-FDG, and ^18^F-FET using dynamic imaging via the Quadra PET system [47]. A recent pilot study achieved total-body perfusion imaging with ^11^C-Butanol on the uEXPLORER system, demonstrating the ability to obtain reproducible measurements of total-body perfusion with the total-body PET system [72].

**Fig. 5:**
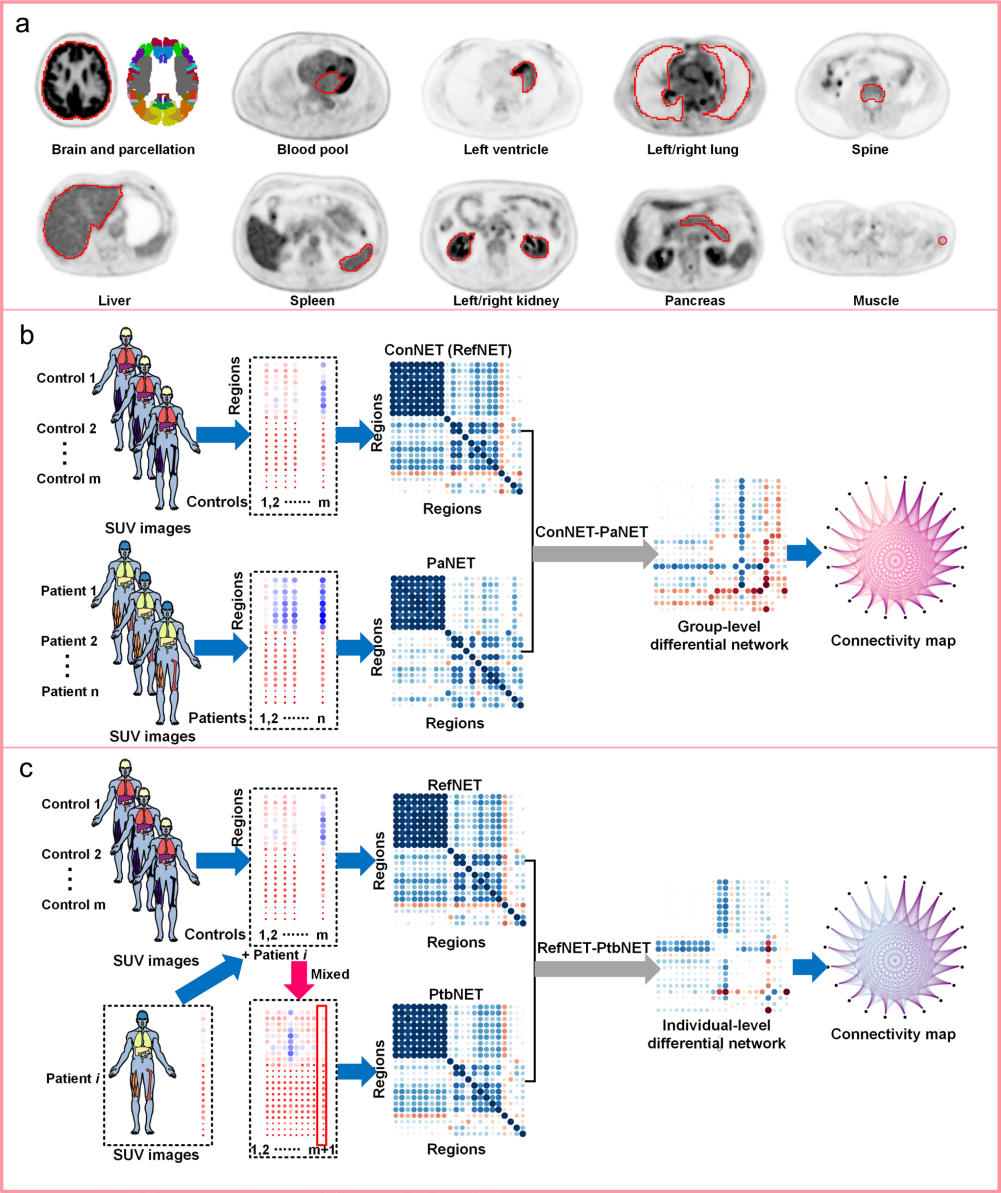
Schematic diagram of the systemic metabolism network constructed via total-body SUV images and partial Pearson correlation coefficients. **a** 18 ROIs including 11 organs and 7 brain regions, according to Sun et al. [63]. **b** Illustration of group-level network analysis. **c** Illustration of individual-level network analysis. Based on the total-body uptake measures (i.e., SUV) images from multi-parameter information obtained by the uEXPLORER, systemic metabolic network can be constructed by calculating the partial Pearson partial correlation coefficient of the uptake measures between the two regions. The group-level differential network is calculated as the difference between the group-level networks of control and patient groups. In terms of individual-level differential network, a reference network of the control group is obtained, then a patient is added to the control group to form a new group, and a network of disturbance connections called the perturbation network is constructed using the same steps. The difference between the perturbation network and the reference network is labeled as the individual-level differential network to describe the deviated metabolic connectivity from the reference network. Abbreviations: RefNET, reference network; ConNET, control group network; PaNET, patient group network; PtbNET, perturbed network

In the past, PET-based studies have mainly focused on regional body parts. The advent of total-body PET systems makes it possible to detect whole-body systemic abnormalities [73]. Recently, Sun et al. developed a network analysis method for ^18^F-FDG total-body PET images (Fig. 5). The developed method has been applied to assess metabolic abnormalities of individuals with lung cancer, gastrointestinal bleeding, and patients after suffering from Covid-19 disease and has revealed metabolic dysfunction from a systemic perspective [63].

The total-body PET systems also have the potential to contribute to the ^89^Zr-antibody imaging exploration of immune diseases [70] and neurodegenerative disorders [74]. In addition, micro-dosing is one of the best approaches in pharmaceutical development since the low concentration of radiopharmaceutical exhibits lower toxicologic risks. The total-body PET/CT system with high sensitivity can provide a more precise estimate of pharmacokinetics and pharmacodynamic [75], contributing to the development of radiopharmaceuticals.

### Clinical application of the total-body PET/CT systems

Compared with performance evaluation and research applications, there were fewer clinical instance for the total-body PET systems. Currently, the main clinical application is in the oncology, and there are three types of tracers used in clinical total-body PET scans, including ^18^F-FDG, ^18^F-Fluciclovine, and ^68^Ga-DOTATATE [42]. For the ^18^F-FDG and ^68^Ga-DOTATATE, according to the experience at University of California, Davis, the recommended scan protocol is 20-min scan at 2 h post-injection with 296 MBq (8 mCi) of ^18^F-FDG or ^68^Ga-DOTATATE. The recommended protocol for the ^18^F-Fluciclovine is 25-min scan at the time of injection with doses ranging from 185 MBq (5 mCi) to 296 MBq (8 mCi) [42].

Total-body PET/CT is also becoming an effective imaging tool in the diagnosis of complicated diseases. Wang et al. revealed the internal relationship of pancreatic disease, pancreatic lipid membrane inflammation and polyarthritis in panniculitis, polyarthritis, and pancreatitis syndrome (PPP) at the molecular imaging level based on the uEXPLORER total-body PET/CT imaging in a patient diagnosed with multiple metastases of pancreatic cancer associated with pancreatic lipid membranitis, avoiding the high mortality associated with delayed diagnosis of PPP [76]. Total-body PET systems also enable accurate assessment of rheumatic immune disease at systemic level and can help to distinguish rheumatoid immune diseases from tumors, lymphomas, or identify the primary tumor lesions that cause arthritic manifestations [77, 78].

### Deep learning

The combination of the total-body PET imaging with machine learning techniques, especially deep learning, may have the potential to solve practical problems that exist in current PET applications [79, 80].

Since analytical reconstruction methods may generate images with a high level of noise, and iterative methods are time-consuming for total-body PET image reconstruction [81], deep learning-based methods have been applied for total-body PET image reconstruction. Ma et al. [81] proposed an encoder-decoder network and achieved reconstruction of total-body PET images on sinograms from the Quadra PET system. Using ultra-high-quality PET images from uEXPLORER, Wang et al. [82] developed a deep progressive learning (DPL) image reconstruction method and verified that the algorithm could reduce the injection dose by 66% without the loss of image quality and even outperformed the traditional algorithm with full dose for small lesion visualization. Yang et al. [83] proposed a DPL method for PET image reconstruction. The network was trained with high-quality PET images from the uEXPLORER and was then incorporated into an iterative reconstruction process, which bridges the gap between low-quality and high-quality images, resulting in better contrast for small lesions.

For tracer kinetic modeling, an image-derived input function (IDIF) is often extracted from an ROI selected within a large blood pool [84, 85]. However, for peripheral tissue region, the arrival time of the radiotracer is delayed compared with the start time of the extracted blood input function [86]. Wang et al. [86] have constructed a deep learning-based regression model that can estimate time delay and/or dispersion of the blood input from the tissue TAC data in combination with the blood input function. In addition, the IDIF represents the tracer activity in the whole blood, a plasma input function is what is actually needed [84, 85]. Deep learning has the potential to predict the input function from tissue TACs. For instance, Kuttner et al. [87] estimated the IDIF from multiple blood pool TACs or multiple tissue TACs using a Gaussian process model and long short-term memory network. Wang et al. [88] used deep learning to estimate IDIF directly from dynamic images instead of from manually placed ROIs.

In terms of parametric imaging, *K*_*i*_ provides equivalent or superior lesion detectability in comparison with SUV [89]. However, parametric imaging takes a long acquisition time to generate *K*_*i*_ images [90]. Several studies have demonstrated that deep learning has the potential to generate parametric images from static PET images and is robust to noise [91, 92]. Huang et al. proposed a fast deep learning method with static SUV images via the uEXPLORER system as the input, and Patlak Ki images as the ground truth. The trained model can generate total-body parametric Ki images without an IDIF [90]. Li et al. [48] configured a deep learning framework called DeepPET and reconstructed a high-quality direct Patlak Ki image from five-frame sinograms via the Quadra system without input function.

In addition, using the powerful mapping ability of deep learning algorithms and the training data from the total-body PET scanners is one way to overcome hardware limitations of short-axis PET scanners [93]. Shang et al. proposed a supervised deep learning model based on a cycle-consistent generative adversarial network (CycleGAN). Trained using data from uEXPLORER, CycleGAN was able to improve the image quality of PET scanners with 320 mm aFOV or even shorter aFOVs [93].

In summary, the introduction of deep learning algorithms not only solved general medical imaging problems including image reconstruction and kinetics modeling [90], but also improved the performances of traditional short-axis PET systems with algorithms specifically designed using training data from the uEXPLORER [82, 83]. However, the existing total-body PET imaging datasets is relatively small, which may limit the development of total-body PET-based deep learning models. With the enrichment of total-body PET datasets in the future, deep learning models based on the total-body PET systems will become more accurate and efficient [86].

## Discussions and conclusions

The total-body PET system, as the most advanced molecular imaging platform for the human body, extends the aFOV to above 1 m [1, 7, 8, 20]. In this review, we have collected and summarized technical reports and studies using the three total-body PET systems in the PubMed database over the past 5 years. Among the three existing systems, the uEXPLORER and Biograph Vision Quadra have been commercially available, and have already been applied in clinical practice, while the PennPET Explorer has not been commercially available.

In general, the hardware upgrade of the three total-body PET systems increases the sensitivity of the systems, allowing for ultrafast [18, 26, 29, 33–36], low-dose [37–41], delayed, and dynamic imaging [18, 46–51]. Due to the upgraded of the total-body PET system, its applications are also expanding, which includes but not limited to the diagnosis of tumor, tumor metastasis, rare diseases, and immunological disorders [35, 39, 40, 54, 64–66, 70–72, 76]. Moreover, based on the total-body PET systems, researchers have developed deep learning models for generating parametric images, and models that can improve image quality of conventional short-axis PET systems [81–83, 86, 90, 93].

The technical advantages of the total-body PET systems enable the applications for many research purposes, well beyond the applications mentioned above. However, most current studies have been focused on the performance evaluation such as reduced scan time or reduced radiopharmaceutical doses, and little attention has been paid to the clinical aspect of the diseases. Specifically, studies on systemic metabolic diseases and tumor metastases, which involve multiple organs and tissues across the human body, are currently available only in few cases mentioned in this review [54, 60, 76]. The 4-dimensional (4D) data acquired from dynamic PET may reflect a broad spectrum of physiological information, including blood flow, tracer delivery, transport, and metabolism [86]. However, current dynamic PET studies via the total-body systems have been limited to single organ analysis, such as the brain and heart [47, 53]. Total-body dynamic imaging studies are still lacking. Future total-body PET imaging studies should pay more attention to on physiological and biologically relevant assays such as cerebral blood volume, blood flow, glucose metabolism, oxygen utilization, DNA synthesis, signal transduction, cancer cell phenotyping for molecularly targeted therapies, pharmacokinetics, pharmacodynamics, etc., to assist in the diagnosis of diseases [94].

In addition, the sample size of existing studies was generally small, and future studies with larger sample sizes are needed. Regarding machine leaning, various deep learning algorithms can effectively improve the performance of total-body PET systems, but the combination of total-body PET systems and deep learning is currently in its infancy due to limited number of total-body PET imaging datasets. Lastly, since total-body PET/CT images can be rendered to show the subject’s face and body, privacy concerns have arisen [95]. Selfridge et al. [95] developed a method that obscures a subject’s face in 3-dimensional volumetric data, which is recommended when sharing total-body PET imaging data.

In conclusion, since living organisms maintain homeostasis through dynamic multi-organ interactions, the total-body PET offers unique opportunities to investigate multi-organ interactions for the understanding of human physiology and pathology [73]. As the prosper of total-body PET-based studies and the enrichment of research data, they can serve as an invaluable tool for elucidating underlying biology across the human body, opening new path for innovative research in physiology, biochemistry, and pharmacology [49]. In precision medicine, the total-body PET systems stand a chance to identify metastases of inflammation, infection and cancer throughout the body and assist doctors to develop precise treatment plans. In drug development, it will enable whole-body pharmacokinetic examinations to observe dynamic uptake of drugs in each organ and tissue in vivo, thus significantly shortening the drug development process. In a word, the total-body PET will certainly push the existing boundaries of PET imaging and make a greater contribution in many medical fields.

## Data Availability

Not applicable.

## Abbreviations

CT: Computed tomography
aFOV: Axial field of view
CNN: Convolutional neural network
CRC: Colorectal cancer
FDG: Fluorodeoxyglucose
PET: Positron emission tomography
SNR: Signal-to-noise ratio
SUL: Standardized uptake value normalized by lean body mass
SUV: Standardized uptake value
TAC: Time-activity curve
